# Structural binding site comparisons reveal Crizotinib as a novel LRRK2 inhibitor

**DOI:** 10.1016/j.csbj.2021.06.013

**Published:** 2021-06-10

**Authors:** Sarah Naomi Bolz, Sebastian Salentin, Gary Jennings, V. Joachim Haupt, Jared Sterneckert, Michael Schroeder

**Affiliations:** a Biotechnology Center (BIOTEC), Technische Universität Dresden, Tatzberg 47/49, Dresden 01307, Germany; b Center for Regenerative Therapies Dresden (CRTD), Technische Universität Dresden, Fetscherstr. 105, Dresden 01307, Germany

**Keywords:** Protein–ligand interactions, Drug repositioning, Binding site, LRRK2, Crizotinib, Structure-based screening

## Abstract

Mutations in leucine-rich repeat kinase 2 (LRRK2) are a frequent cause of autosomal dominant Parkinson’s disease (PD) and have been associated with familial and sporadic PD. Reducing the kinase activity of LRRK2 is a promising therapeutic strategy since pathogenic mutations increase the kinase activity. Several small-molecule LRRK2 inhibitors are currently under investigation for the treatment of PD. However, drug discovery and development are always accompanied by high costs and a risk of late failure. The use of already approved drugs for a new indication, which is known as drug repositioning, can reduce the cost and risk.

In this study, we applied a structure-based drug repositioning approach to identify new LRRK2 inhibitors that are already approved for a different indication. In a large-scale structure-based screening, we compared the protein–ligand interaction patterns of known LRRK2 inhibitors with protein–ligand complexes in the PDB. The screening yielded 6 drug repositioning candidates. Two of these candidates, Sunitinib and Crizotinib, demonstrated an inhibition potency (IC50) and binding affinity (K_d_) in the nanomolar to micromolar range. While Sunitinib has already been known to inhibit LRRK2, Crizotinib is a novel LRRK2 binder.

Our results underscore the potential of structure-based methods for drug discovery and development. In light of the recent breakthroughs in cryo-electron microscopy and structure prediction, we believe that structure-based approaches like ours will grow in importance.

## Graphical abstract

**Figure:**
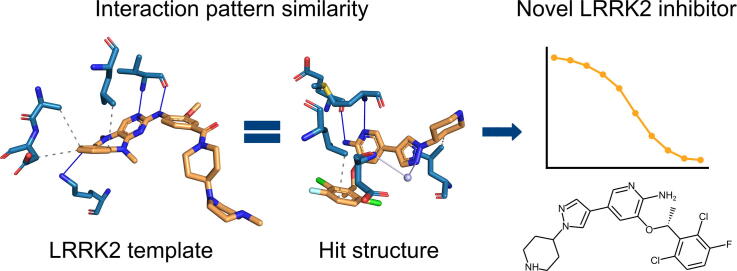

## 1 Background

Parkinson’s disease (PD) is a common neurological disorder that affected 6.1 million people and caused 211,296 deaths in 2016 alone [1]. The disease is characterized by the loss of dopaminergic neurons and the presence of aberrant protein aggregates of *α*-synuclein in the brain. In addition to the typical parkinsonian motor symptoms, patients suffer from non-motor symptoms like cognitive impairment, mental illness, and olfactory dysfunction [2]. The risk of developing PD is thought to be determined by an interplay of both environmental factors and genetics [3]. A frequent cause of autosomal dominant forms of the disease are mutations in the leucine-rich repeat kinase 2 (LRRK2) [2], [4]. Mutations in LRRK2 are found in patients with familial PD and also have been implicated with sporadic PD [5]. G2019S is the most common known pathogenic LRRK2 variant and shows increased kinase activity. Blocking of LRRK2 kinase activity using small-molecule inhibitors has neuroprotective effects in some PD models [6]. At the same time, a partial loss of function of LRRK2 does not appear to lead to a particular phenotype or disease in humans [7]. This makes the reduction of LRRK2 kinase activity a promising and presumably safe therapeutic strategy for PD. Several small-molecule inhibitors that target LRRK2 are currently under investigation and some are close to entering clinical trials [8]. However, drug discovery and development is an expensive process [9], [10] that is at risk of late failure due to poor efficacy or severe side effects revealed during clinical trials [11].

Drug repositioning, which is the use of already approved drugs for new indications, has the potential to reduce the cost, time, and risk of drug development since many biochemical and clinical factors, such as safety or adsorption, are already known for approved drugs [12]. Candidates for drug repositioning can be systematically identified. One strategy, also known as drug-centric approach, is to connect a known drug to a new target that is related to a different indication [13]. The exponential growth of structural data [14] and recent progress in protein structure prediction [15], [16] make structure-based methods a promising approach to this strategy [17]. As a prime example, we recently identified three potent, inexpensive, and easily available drug repositioning candidates for Chagas disease by comparing protein–ligand interaction profiles of Chagas targets and other proteins [18].

**Fig. 1:**
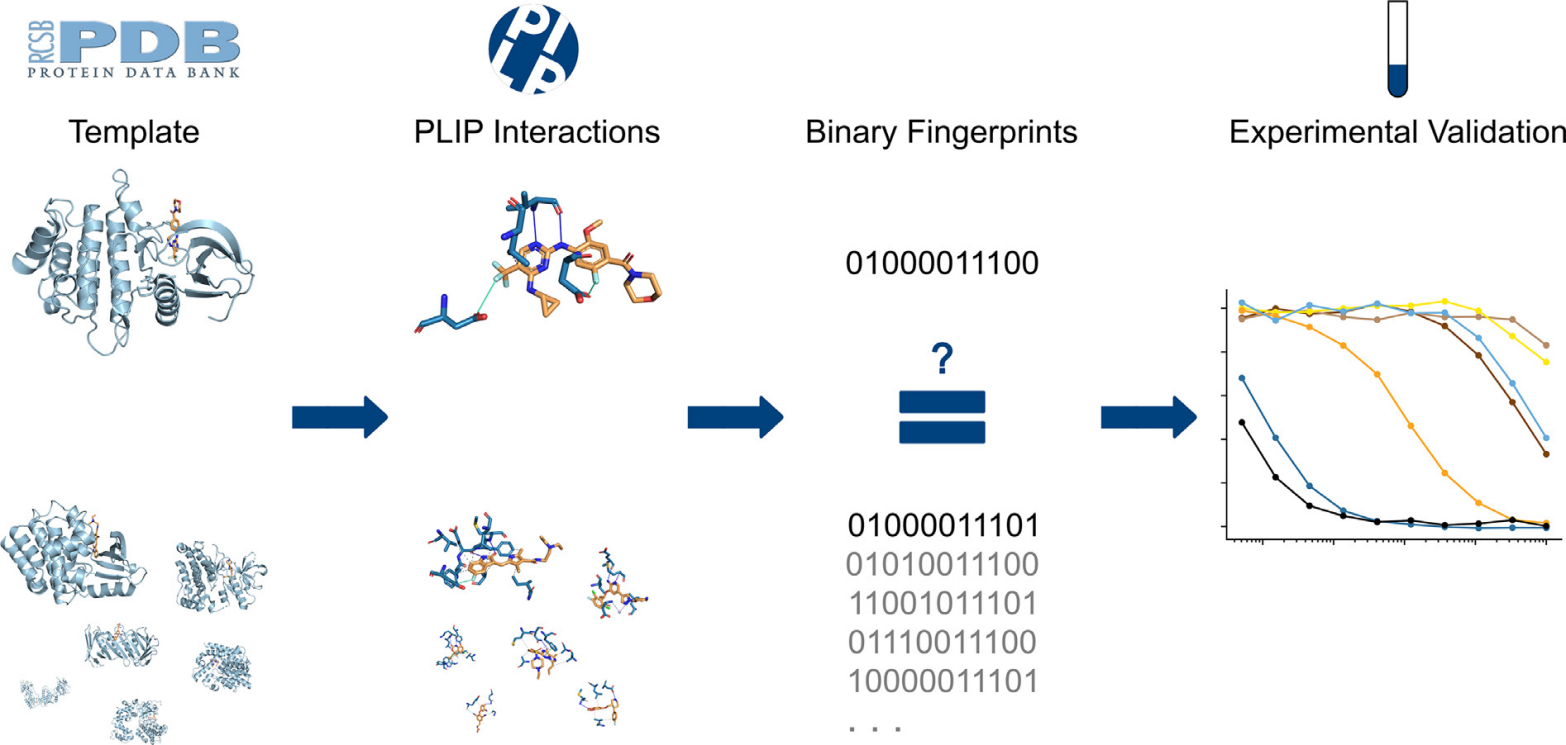
Overview over the study. PLIP interactions are extracted from a LRRK2 template structure with inhibitor. They are encoded as a fingerprint, which is compared to each and every fingerprint in PDB. The top matches are experimentally validated.

Here, we use a structure-based drug repositioning approach to identify new inhibitors of LRRK2. Our approach is based on the observation that drugs bind different targets with similar interaction patterns. To identify new LRRK2 inhibitors, we determine the interaction pattern of a LRRK2 template structure with inhibitor using the Protein–Ligand Interaction Profiler (PLIP) [19], [20] and compare it to the interaction patterns of all complex structures in the Protein Data Bank (PDB) [21]. We use binary interaction fingerprints to represent the interaction patterns of the template and PDB structures (Fig. 1). The top matches of the screening are manually filtered and experimentally validated.

A prerequisite for our approach is the availability of a protein–ligand complex structure of the query target. With multiple structurally diverse domains and a molecular weight six times higher than the average 30–60 kDa, LRRK2 is a difficult to crystallize protein. High-quality structural information on the protein currently only includes lower organism homologs or parts of the protein like single domains [22], [23], [24], [25], [26], [27], [28]. Recently, some cryo-electron microscopy structures with coordinates for large parts of LRRK2 have been released in the PDB (PDB IDs:  6VNO, 6VP6, 6VP7, 6VP8, and 6XR4 [22], [23]). Yet, there is no protein–ligand complex structure of LRRK2 available in the PDB. To overcome this lack of information, we selected complex structures of two LRRK2 inhibitors bound to the humanized LRRK2 homolog Roco4 as templates.

## 2 Methods

### 2.1 Virtual screening and characterization of hit compounds

We retrieved complex structures that served as queries for the virtual screening from the PDB. Since there was no LRRK2 complex available in the PDB, complex structures of two LRRK2 inhibitors bound to the humanized LRRK2 homolog Roco4 (PDB IDs:  4YZM and  4YZN [27]) were used.

We determined the non-covalent interactions between the inhibitors and Roco4 using the Protein–Ligand Interaction Profiler (PLIP) [19], [20]. The interaction pattern of each inhibitor was then encoded into an interaction fingerprint, a binary vector in which each bin represents an interaction feature. An interaction feature was defined by the combination of two non-covalent interactions within an angle and distance range. If a particular interaction feature was present in the interaction pattern, the respective bin was set to 1, otherwise, it was set to 0, as already described in [29], [18]. The interaction fingerprints are available from PharmAI (Dresden, Germany).

Subsequently, the Tanimoto similarity of the query interaction fingerprints to the fingerprints of all protein–ligand complexes in the PDB was determined. The screening was performed on the full PDB without prior filtering. The Tanimoto similarity is calculated from the intersection of on-bins between fingerprint A and B divided by the sum of on-bins in A and on-bins in B minus the intersection of on-bins between A and B. The complexes were ranked according to the overall similarity of their interaction patterns to the query. Complexes that showed an empirical p-value of 0.001 (based on the distribution of all pairwise similarities) or less (Fig. A.1) and contained an FDA-approved drug were considered hits.

Hit complexes were manually filtered according to two criteria: 1) agreement of key interaction features after visual inspection and 2) drugs with adverse-effects considered too severe for treatment of Parkinson’s disease patients or compounds that were considered to be inappropriate for systemic exposure were discarded.

The SwissADME web service [30] was used to assess physicochemical properties of the six candidate drugs identified by interaction-based virtual screening. The BOILED-Egg algorithm [31] integrated into the web service calculates the lipophilicity and polarity of molecular structures to predict gut epithelial and blood/brain barrier (BBB) permeation. Literature was used to confirm the BBB permeability predictions.

To calculate the chemical similarity between compounds, canonical smiles were retrieved from PubChem [32]. We used the *Chem.AllChem.GetMorganFingerprintAsBitVect()* method from RDKit [33] to generate circular Morgan fingerprints with 16384 bits and a diameter of 4. The Morgan fingerprints correspond to extended-connectivity fingerprints as described in [34], [35]. The Tanimoto similarity was used as fingerprint similarity metric. The chemical similarity heatmap was generated using the python package seaborn [36] (version 0.11.1) with the *heatmap()* method.

### 2.2 Sequence and structural similarity of humanized Roco4 and LRRK2

For sequence identity calculation, the amino acid sequences of Roco4 and LRRK2 were downloaded from UniProt [37]. To get the humanized sequence of Roco4, the two phenylalanine residues Phe1107 and Phe1161 were manually modified to leucines [27]. Global sequence identity was calculated using the EMBOSS Needle Pairwise Sequence Alignment web service with default parameters [38]. To analyze the structural similarity, we selected a cryo-electron microscopy structure of LRRK2 (PDB ID: 6VNO [23]) and structurally aligned it with both chains of the Roco4 LRRK2-IN-1 complex (PDB ID: 4YZM) and with the Roco4 Compound19 complex (PDB ID: 4YZN). PyMOL [39] (version 2.4.0) with the *super* command was used for structural alignment of the proteins and RMSD (root-mean-square deviation of atomic positions) determination.

### 2.3 Experimental validation

The six hit compounds from the virtual screening were ordered from Selleckchem (Houston, Texas, USA). GW 5074 was ordered from Tocris Bioscience (Bristol, UK). Stocks of all compounds were shipped to Reaction Biology Corporation (Malvern, Pennsylvania, USA) and Eurofins DiscoverX (Fremont, California, USA) for IC50 and K_d_ determination, respectively. The IC50 and K_d_ assays were performed as service by the companies.

For IC50 measurements, compounds were tested at 10 different concentrations in duplicates with 3-fold serial dilution starting at 100 μM. Compounds were dissolved and diluted in 100% DMSO. Briefly, LRRK2 G2019S and a peptide substrate ([RLGRDKYKTLRQIRQ]) were prepared in reaction buffer; 20 mM Hepes (pH 7.5), 10 mM MgCl_2_, 1 mM EGTA, 0.02% Brij35, 0.02 mg/ml BSA, 0.1 mM Na_3_VO_4_, 2 mM DTT, 1% DMSO. Compounds were delivered into the reaction mixture. After a 20 min incubation, ^33^P-ATP was added to a final concentration of 10 μM to initiate the reaction. Reactions were carried out for 2 h at room temperature. The final enzyme and substrate concentrations were 20 nM and 20 μM, respectively. Kinase activity was detected by the P81 filter-binding method. The whole procedure is also described in [40].

K_d_ values were determined via the KINOMEscan assay, which is based on a competition binding assay that quantitatively measures the ability of a compound to compete with an immobilized, active-site directed ligand. The compounds were tested at 11 different concentrations in duplicates with 3-fold serial dilution starting at 100 μM. Compounds were dissolved and diluted in 100% DMSO. LRRK2 G2019S was tagged with DNA for qPCR detection. Shortly, streptavidin-coated magnetic beads were treated with the biotinylated competitive ligand for 30 min at room temperature. The liganded beads were blocked with excess biotin and washed with blocking buffer (SeaBlock (Pierce), 1% BSA, 0.05% Tween 20, 1 mM DTT) to remove unbound ligand and to reduce non-specific binding. Binding reactions were assembled by combining kinase, liganded affinity beads, and test compounds in 1x binding buffer (20% SeaBlock, 0.17x PBS, 0.05% Tween 20, 6 mM DTT). The assay plates were incubated at room temperature with shaking for 1 h and the affinity beads were washed with wash buffer (1x PBS, 0.05% Tween 20). The beads were then re-suspended in elution buffer (1x PBS, 0.05% Tween 20, 0.5 μM non-biotinylated affinity ligand) and incubated at room temperature with shaking for 30 min. The kinase concentration in the eluates was measured by qPCR.

## 3 Results

### 3.1 Virtual screening

To identify repositioning candidates for the inhibition of LRRK2, we performed a virtual screening that identifies potential binders based on interaction pattern similarity. We chose structures of the humanized LRRK2 homolog Roco4 in complex with two LRRK2 inhibitors (PDB IDs: 4YZM and 4YZN  [27]) as query structures for the screening because there was no LRRK2 complex structure available in the PDB.

**Fig. 2:**
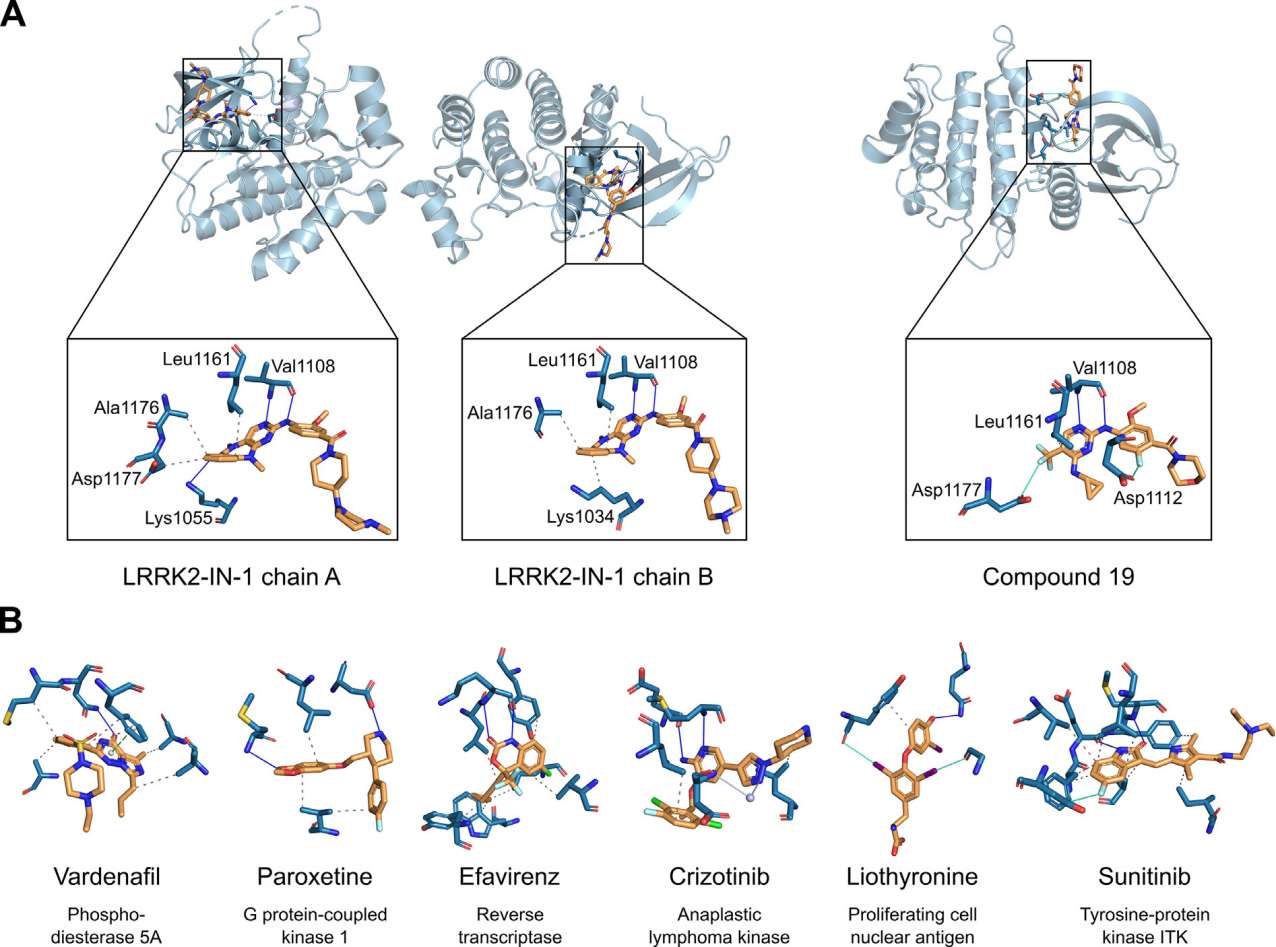
Protein–ligand interactions detected by PLIP of the LRRK2 inhibitors and the hit compounds from the virtual screening. Protein residues are shown in blue and compounds in orange. (A) Two structures of humanized Roco4 with LRRK2-IN-1 (left, PDB ID: 4YZM) and Compound19 (right, PDB ID: 4YZN). LRRK2-IN-1 interacts with two chains of Roco4. Above is the cartoon representation of Roco4 in complex with the inhibitors. Below is a close-up of the interactions between the inhibitors and and the binding site residues of Roco4. (B) Interactions between the hit compounds and their targets. All compounds share key interaction features with one of the LRRK2 inhibitors.

Non-covalent interactions between the inhibitors and Roco4 were detected using PLIP (Fig. 2A). Both inhibitors are anchored to the binding site by two hydrogen bonds with the backbone of Val1108. The interaction pattern of Compound19 [41] is furthermore defined by two halogen bonds and a hydrophobic interaction with the side-chains of Asp1177, Asp1112, and Leu1161, respectively. LRRK2-IN-1 [42] interacts with two chains of Roco4. In chain A, LRRK2-IN-1 forms another hydrogen bond with Lys1055 and contacts the side-chains of Leu1161, Ala1176, and Asp1177 via hydrophobic interactions. In chain B, the inhibitor does not interact with Asp1177 and Lys1055, but forms a hydrophobic interaction with Lys1034.

**Fig. 3:**
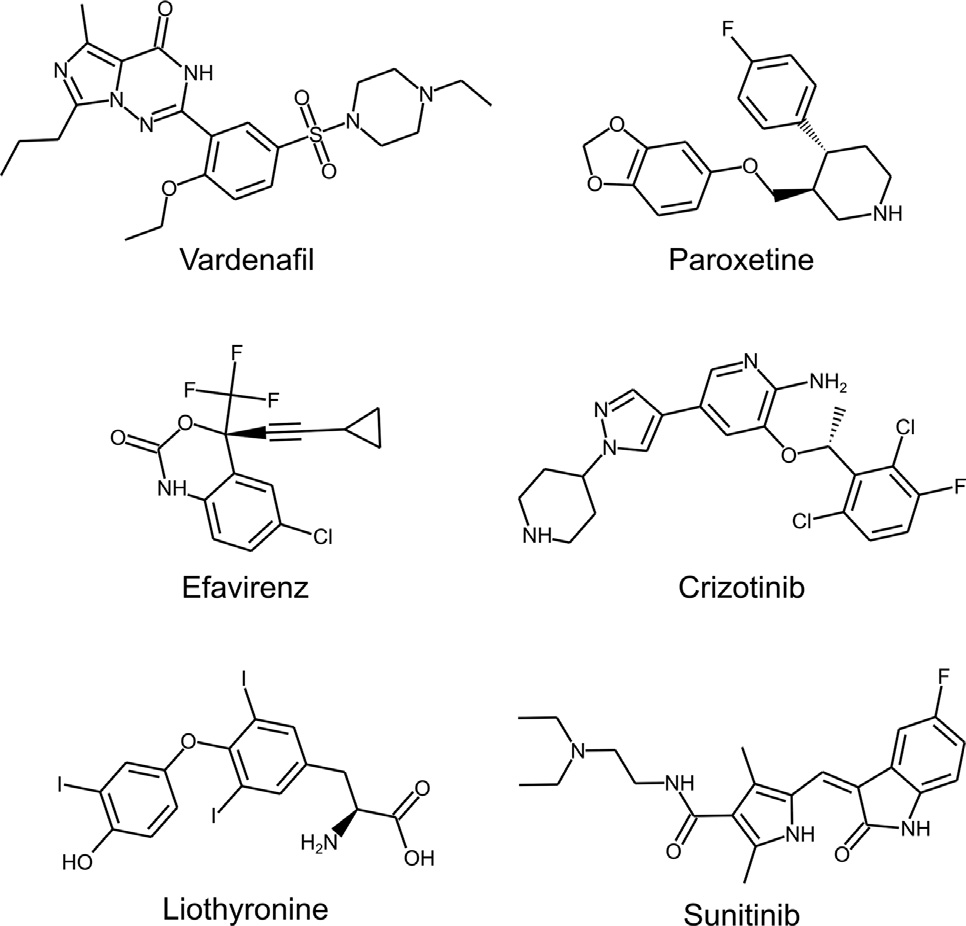
Chemical structures of the six top repositioning candidates identified by the virtual screening.

We encoded the non-covalent interaction patterns into binary interaction fingerprints in which each bin represents a particular interaction feature. We compared the interaction fingerprints of the query structures to the interaction fingerprints of complex structures in the PDB. From the complexes with a significantly similar interaction pattern by p-value, we selected those that contain FDA-approved drugs. This resulted in a total of 15 hit complexes comprising 13 distinct FDA-approved drugs (Table A.1). After manual assessment and filtering involving visual inspection of the complexes and careful evaluation of the compound applicability (Table A.1), 6 top candidates remained (Table 1, Fig. 3).

**Table 1:** Queries and top hits of the virtual structure-based screening. The respective PDB IDs are shown in parentheses. The approved use and the BBB permeability of the hit compounds are given. The query structure with LRRK2-IN-1 yielded four top hits, while the query structure with Compound19 yielded two top hits.

Query	Hit	Approved Use	BBB Permeable
Humanized Roco4 with LRRK2-IN-1 (4YZM)	Human phosphodiesterase 5A with Vardenafil (1XP0)	Vardenafil: inhibitor of phosphodiesterase 5A (in erectile dysfunction)	Yes
	Bovine GPCR kinase with Paroxetine (4L9I)	Paroxetine: serotonin uptake inhibitor (in depression); GPCR Kinase 2 inhibitor	Yes
	HIV-1 reverse transcriptase with Efavirenz (1IKW)	Efavirenz: Non-nucleoside inhibitor of reverse transcriptases (in HIV/AIDS)	Yes
	Mutant ALK with Crizotinib (4ANS)	Crizotinib: double mutant anaplastic lymphoma kinase inhibitor (in oncology)	No
Humanized Roco4 with Compound19 (4YZN)	Proliferating cell nuclear antigen with Liothyronine (3VKX)	Liothyronine: Synthetic thyroid hormone (in hypothyroidism)	Yes
	IL-2-inducible T cell kinase with Sunitinib (3MIY)	Sunitinib: RTK inhibitor (in oncology); known inhibitor of LRRK2	Yes

### 3.2 Humanized Roco4 as LRRK2 template

**Fig. 4:**
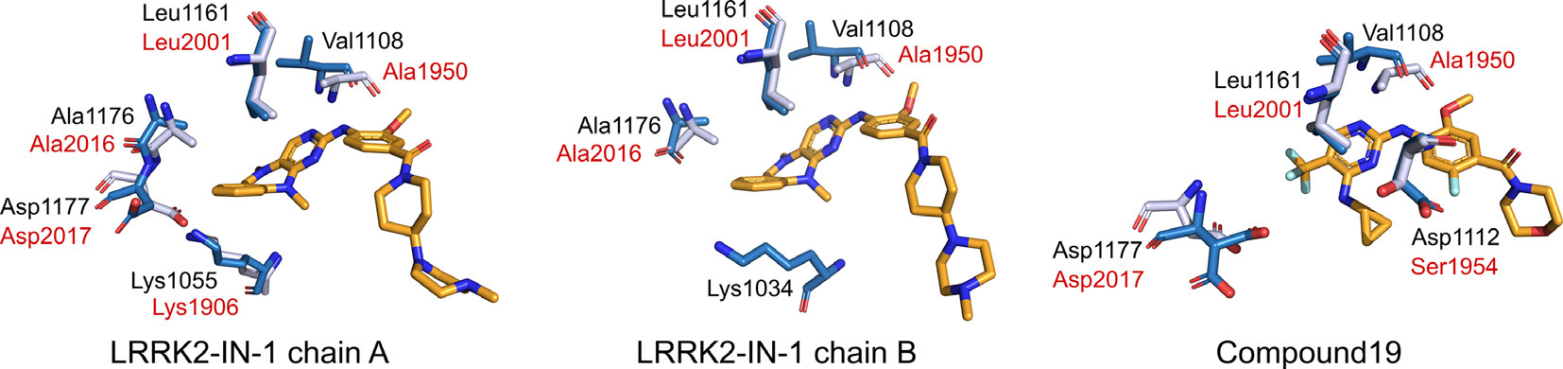
Structural alignment of the interacting residues of humanized Roco4 with the corresponding LRRK2 residues. The alignment is shown for each of the Roco4 query structures from the virtual screening: Roco4 in complex with LRRK2-IN-1 (left and middle, PDB ID: 4YZM) and with Compound19 (right, PDB ID: 4YZN). Compounds are shown in orange. Roco4 residues are illustrated in dark, LRRK2 residues in light blue. Protein residue labels are in black for Roco4 and in red for LRRK2.

Our virtual screening was based on structures of the humanized LRRK2 homolog Roco4. To assess the suitability of humanized Roco4 as a model for LRRK2, we analyzed sequence and structural similarity of the two proteins. Gilsbach et al. achieved humanization of Roco4 by mutating the two phenylalanine residues Phe1107 and Phe1161, which are located at the binding site of the inhibitors, to leucines [27]. Interestingly, the global sequence identity between humanized Roco4 and LRRK2 is only 13%. However, the proteins share significant structural similarity. We structurally aligned the query structures of our virtual screening (PDB IDs: 4YZM and 4YZN [27]) with a cryo-electron microscopy structure of LRRK2 (PDB ID: 6VNO [23]). The RMSDs were 1.74 Å, 1.67 Å, and 1.74 Å for chain A and chain B of Roco4 in complex with LRRK2-IN-1 and Roco4 in complex with Compound19, respectively. The interacting residues of humanized Roco4 align very well with the corresponding residues of the LRRK2 kinase domain (Fig. 4). In chain A of the LRRK2-IN-1 Roco4 complex, Leu1161 aligns with Leu2001, Ala1176 with Ala2016, Asp1177 with Asp2017, and Lys1055 with Lys1906. Moreover, the backbone of Val1108 aligns with the backbone of Ala1950. In chain B of the LRRK2-IN-1 Roco4 complex, we see the same alignment pattern for Leu1161, Ala1176, and Val1108. Lys1034 does not align with any residue of LRRK2. The interacting residues of the Compound19 Roco4 complex are also in good agreement with the respective LRRK2 residues: Leu1161 matches with Leu2001, Asp1177 with Asp2017, and the backbone of Val1108 with the backbone of Ala1950. Asp1112 aligns with Ser1950, which has different side chain properties.

### 3.3 Characterization of the top candidates

Each of the 6 hit compounds shares key interaction features with one of the LRRK2 inhibitors (Fig. 2). Vardenafil and LRRK2-IN-1 both form a hydrogen bond in the central part of the ligand, and the hydrophobic interactions to the benzene ring of Vardenafil match the distance and angle range of the hydrophobic interactions that LRRK2-IN-1 forms with chain A of Roco4. Paroxetine forms two hydrogen bonds at different parts of the ligand. This pattern is also present in the binding mode of LRRK2-IN-1. Moreover, the hydrophobic interactions of the 1,3-Benzodioxole moiety of Paroxetine match the hydrophobic interaction pattern that LRRK2-IN-1 constitutes with Lys1034 and Leu1161 of Roco4 chain B. The interaction pattern of Efavirenz exhibits two parallel hydrogen bonds, which are also present in the interaction patterns of both LRRK2 inhibitors. Although Efavirenz registered as a hit based on its interaction pattern similarity to LRRK2-IN-1, it also matches very well with Compound19 since the halogen atoms are in the same distance range. The case is similar for Crizotinib. The interaction pattern of this compound was found to be significantly similar to the interaction pattern of LRRK2-IN-1, which is most obviously reflected in the parallel hydrogen bonds. Nevertheless, Crizotinib also goes very well with Compound19 as it has halogen atoms in the right position. Liothyronine forms a trinity of two halogen bonds and one hydrogen bond. This pattern can also be found in the interaction pattern of Compound19. Lastly, the interaction pattern of Sunitinib includes two hydrogen bonds and one halogen bond that are in the same distance range as the hydrogen bonds and the halogen bond that Compound19 forms with Val1108 and Asp1177 of Roco4.

The original indications of the top candidates are various, comprising cancer, depression, erectile dysfunction, HIV/AIDS, and hypothyroidism (Table 1). Half of the compounds are known to be kinase inhibitors. Interestingly, there is previous evidence of inhibitory activity against LRRK2 for one of the top candidates: The anti-cancer drug Sunitinib is a receptor tyrosine kinase inhibitor that has been shown to efficiently inhibit LRRK2 [43]. The protein kinase inhibitor Crizotinib is currently approved for use in non-small cell lung carcinoma [44]. Paroxetine is a selective serotonin uptake inhibitor already used for treating depression in Parkinson’s disease [45]. Moreover, it has been identified as a direct inhibitor of G protein-coupled receptor kinase 2 (GRK2), which is a serine/threonine kinase like LRRK2 [46]. Vardenafil is an inhibitor of phosphodiesterase 5 and is applied for the treatment of erectile dysfunction [47]. Liothyronine is a synthetic form of the thyroid hormone triiodothyronine that is used for hypothyroidism [48]. It has been shown that there is an association of low thyroid hormone levels with motor symptoms in Parkinson’s disease [49]. Efavirenz is a non-nucleoside inhibitor of reverse transcriptases and is used for the treatment and prevention of HIV/AIDS [50].

Drugs that target LRRK2 in Parkinson’s disease must be able to cross the BBB in order to reach the brain. Therefore, we predicted BBB permeability for our 6 top candidates using the SwissADME web service. Sunitinib, Paroxetine, and Efavirenz were predicted to penetrate the BBB, while Crizotinib, Vardenafil, and Liothyronine were predicted not to permeate the barrier. The BBB permeability of Sunitinib, Paroxetine, and Efavirenz was supported by literature [51], [52], [53]. In addition, we found evidence in the literature that Vardenafil and Liothyronine cross the BBB [54], [55], while BBB penetration of Crizotinib is poor [56] (Table 1).

**Fig. 5:**
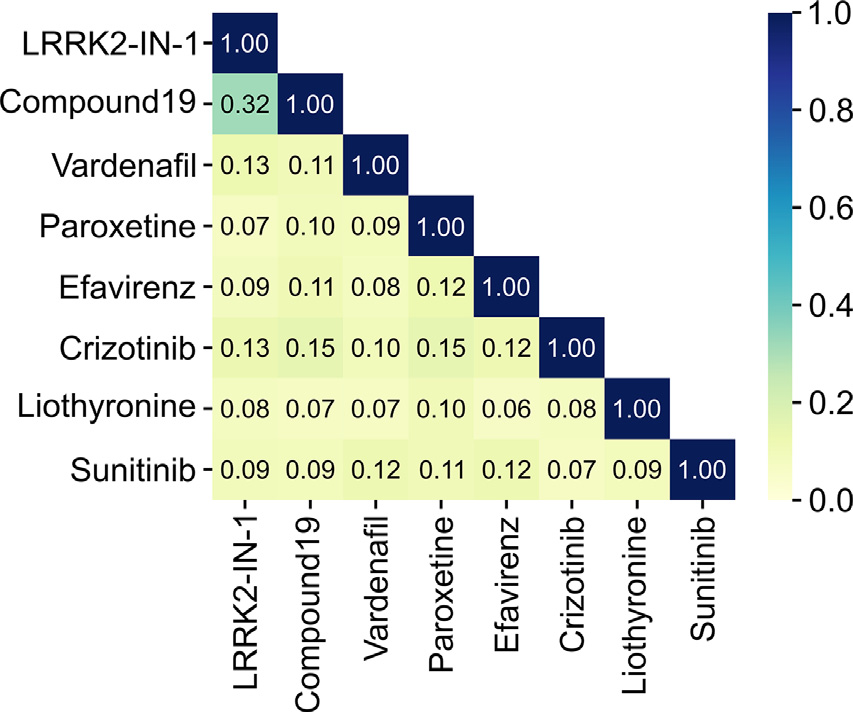
Chemical similarity heatmap of the query and the top hit compounds identified by the virtual screening. The highest chemical similarity is between LRRK2-IN-1 and Compound19.

In contrast to a chemical similarity approach, our interaction pattern similarity approach has the potential to reveal scaffolds that are different to the query compounds. To investigate whether the virtual screening yielded novel scaffolds, we calculated chemical similarities between the query and the hit compounds (Fig. 5). The highest chemical similarity was detected for the two query compounds LRRK2-IN-1 and Compound19. Strikingly, none of the six top repositioning candidates showed a considerable chemical similarity with the query compounds. The same applies to the hit compounds with one another.

### 3.4 Experimental validation of the hit compounds

A specific challenge to our drug repositioning approach was the use of a homolog structure as template instead of a LRRK2 structure. To validate the virtual screening results, all six top candidates were tested for in vitro inhibition of LRRK2 kinase activity. The most common pathogenic LRRK2 mutation in PD is G2019S and is located in the protein kinase domain of LRRK2 [8]. We used LRRK2 G2019S together with an artificial substrate to measure the ability of the hit compounds to inhibit the LRRK2 kinase variant (IC50 determination). Furthermore, we tested the compounds for competitive binding to the active site of the mutant LRRK2 kinase (Kd determination).

**Fig. 6:**
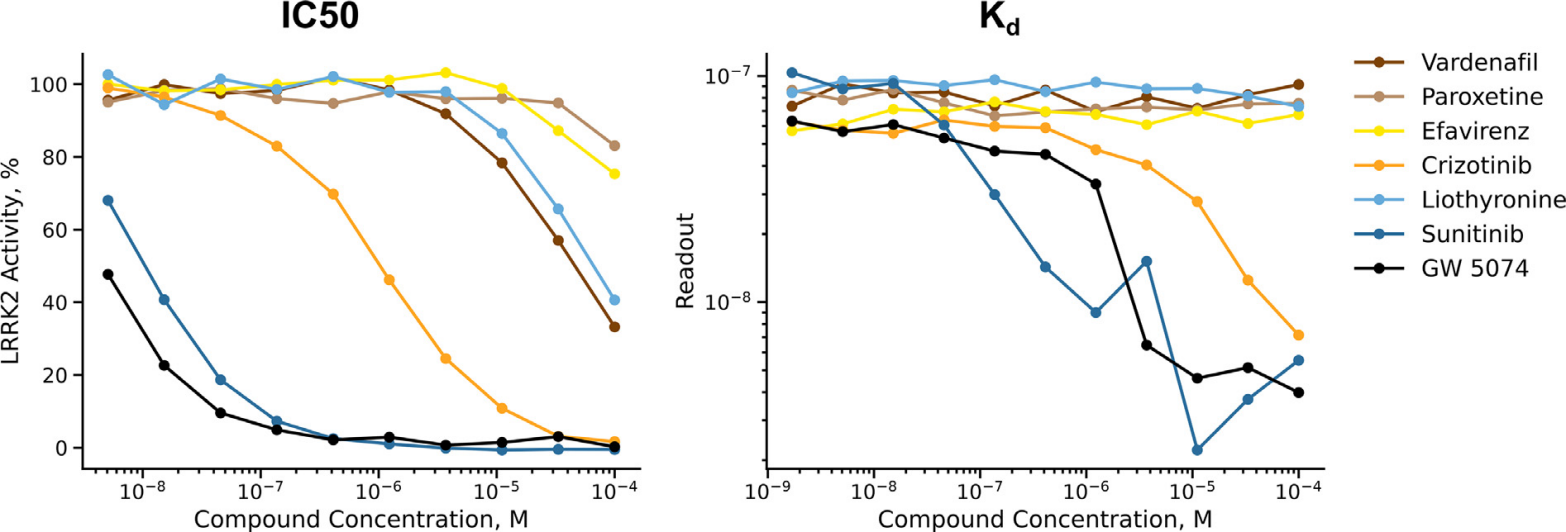
In vitro inhibition of (left, IC50) and binding to (right, Kd) LRRK2 G2019S by the six top candidates from the virtual screening. GW 5074 served as positive control. In the IC50 plot, LRRK2 activity is plotted against the respective compound concentration. In the Kd plot, the readout is plotted against the compound concentration. The Kd assay measured the ability of the hit compounds to compete with an active-site directed ligand. The readout was the amount of LRRK2 bound to the competitive ligand. Both assays were performed in duplicates and plots show average values. Sunitinib and Crizotinib have an IC50 and Kd in the micromolar to nanomolar range.

Four of the six top candidates demonstrated an IC50 below 100 μM (Table 2, Fig. 6). The measured IC50 values were 10 nM, 1 μM, 47 μM, and 67 μM for Sunitinib, Crizotinib, Vardenafil, and Liothyronine, respectively. Of these four compounds, Sunitinib and Crizotinib produced a K_d_ less than 100 μM. While Crizotinib showed a K_d_ of 8 μM, Sunitinib produced a K_d_ of 67 nM.

**Table 2:** IC50 and K_d_ of the top candidate drugs. GW 5074 was the positive control. Sunitinib and Crizotinib showed IC50 and K_d_ values in the micromolar to nanomolar range.

Compound	IC50 (nM)	K_d_ (nM)
Vardenafil	470,00	>100,000
Paroxetine	>100,000	>100,000
Efavirenz	>100,000	>100,000
Crizotinib	980	8300
Liothyronine	67000	>100,000
Sunitinib	10	67
GW 5074	<5.1	790

## 4 Discussion and conclusion

LRRK2 is considered a promising therapeutic target in Parkinson’s disease. Using a structure-based drug repositioning approach, we identified two approved cancer drugs that inhibit LRRK2 with an IC50 and a K_d_ in the nanomolar to micromolar range.

Sunitinib is a multi-target tyrosine kinase inhibitor that modulates the activity of several kinases, including vascular endothelial growth factor receptors (VEGFRs), platelet-derived growth factor receptors (PDGFR), stem cell factor receptor (KIT), and fms-like tyrosine kinase 3 (FLT3). It competitively binds the kinases at the ATP binding site [57]. Sunitinib is approved for the treatment of different cancers, such as metastatic renal cell carcinoma (mRCC) [58] and pancreatic neuroendocrine tumors (pNET) [59]. Consistent with previous reports [60], [43], we found that Sunitinib is a potent inhibitor and binder of LRRK2 G2019S. The retrieval of a known binder serves as a good validation of our virtual screening approach.

Crizotinib is a tyrosine kinase inhibitor that targets hepatocyte growth factor receptor (HGFR), anaplastic lymphoma kinase (ALK), and proto-oncogene tyrosine-protein kinase ROS (ROS1) through ATP-competitive binding. It is approved for use in non-small cell lung cancer (NSCLC) as an inhibitor of the mutant form of ALK [44]. We showed that Crizotinib is a moderate inhibitor and binder of LRRK2 and to our knowledge, there are no previous reports of this pharmacology published in the scientific literature. In contrast to Sunitinib, Crizotinib is not brain penetrant. However, given that brain metastases are a common and lethal complication of NSCLC and Crizotinib-treated NSCLC patients eventually develop resistance, the brain penetrant ALK inhibitor Lorlatinib, a cyclized derivative of Crizotinib, has been developed [61]. The drug has been approved for the treatment of ALK-positive metastatic NSCLC in 2018 [62]. Our results suggest that Lorlatinib could also be a LRRK2 kinase inhibitor useful in the treatment of Parkinson’s disease.

Sunitinib and Crizotinib are chemically dissimilar from the query compounds LRRK2-IN-1 and Compound19. Moreover, Crizotinib has a negligible chemical similarity with the known LRRK2 binder Sunitinib. This proves that our interaction pattern similarity approach is capable of identifying novel scaffolds that would not have been revealed by chemical similarity approaches.

Our structure-based approach was challenged by the lack of LRRK2 complex structures. We used complex structures of the humanized LRRK2 homolog Roco4 instead. Overall, humanized Roco4 and LRRK2 have little sequence identity but display high structural similarity. In particular, the binding site residues of humanized Roco4 align very well with the corresponding residues of the LRRK2 kinase domain. This underscores the suitability of Roco4 as model for LRRK2. Using this model, we yielded a surprisingly good success rate. Out of six top candidates identified by our virtual screening, two effectively bind and inhibit LRRK2 G2019S. It should be noted that, strictly speaking, virtual screening and experimental validation do not measure the same parameters. The virtual screening captures the interaction similarity between query and hit complexes. However, to manifest these interactions, compounds and proteins must overcome many physicochemical constraints, such as assay conditions, access to the protein binding site, potential compound tautomerization, and non-specific protein–protein, compound-compound, and protein-compound binding. These effects largely influence in vitro binding kinetics (K_d_) and the potency of inhibition (IC50).

In high-throughput screenings, thousands or millions of compounds are tested against the target of interest to identify novel binders. In contrast, we created a focused chemical library with only six test compounds using our structure-based screening approach. The compounds identified by our screening are more likely to bind than a random selection of compounds because their interaction patterns are similar to those of known binders. However, the approach is more of a statistical nature and cannot perfectly predict binding. Therefore, experimental validation is key to determining the true binders. Moreover, our approach cannot be compared to a quantitative structure–activity relationship (QSAR) study in which details of an interaction can be interpreted to inform the next steps in drug development. Rather, the method should be seen as a step before a QSAR study. Another limit of the virtual screening is that it merely predicts binding and not success in follow-up studies such as cell assay and *in vivo* studies. Still, as a screening approach, our structure-based method has very high success rates. In previous studies, the approach demonstrated to predict 5–10% binders [63], [29], [18], which is superior to high-throughput screenings. In the present study, two out of six hit compounds actually bind LRRK2 G2019S, which corresponds to a success rate of 33%.

As with all structure-based approaches, the feasibility and success of our computational drug repositioning screening depend on the availability of structural data. Despite the rapid growth of structural data, the number of compounds in the PDB is tiny compared to chemical databases like ChEMBL [64]. However, this could change dramatically in the near future. Recent advances in cryo-electron microscopy now allow researchers to obtain high-resolution structures of proteins that are difficult to crystallize [65]. In addition, structure prediction has recently made a major breakthrough when the artificial intelligence system AlphaFold developed by DeepMind achieved unprecedented accuracy in the Critical Assessment of protein Structure Prediction (CASP) [16]. These advances will increase the importance of structure-based methods like our virtual drug repositioning approach.

To conclude, the structure-based drug repositioning approach presented in this work has proven to be successful in identifying LRRK2 kinase inhibitors. With the virtual screening, we were able to retrieve a known LRRK2 binder and to find a novel binder. We showed that the LRRK2 homolog Roco4 served as a sufficient query for the screening. Our results demonstrate the power and potential of structure-based drug repositioning, which will gain in importance in the next years.

## Funding

This work was supported by the 10.13039/501100002347Federal Ministry of Education and Research, Germany (project Redivia, Grant No. 03EFISN108). The funding source was not involved in study design, collection, analysis, and interpretation of data, writing of the report, or the decision to submit the article for publication.

## CRediT authorship contribution statement

**Sarah Naomi Bolz:** Writing - original draft, Writing - review & editing, Formal analysis, Visualization. **Sebastian Salentin:** Writing - review & editing, Formal analysis, Software. **Gary Jennings:** Conceptualization, Validation. V. Joachim Haupt: Formal analysis. **Jared Sterneckert:** Validation. **Michael Schroeder:** Conceptualization, Formal analysis, Supervision, Writing - review & editing, Visualization.

## Conflict of interest

The authors declare that they have no known competing financial interests or personal relationships that could have appeared to influence the work reported in this paper.
