# Supplementary material for: Structural binding site comparisons reveal Crizotinib as a novel LRRK2 inhibitor
Source: Comput Struct Biotechnol J. 2021 Jun 10;19:3674–81. doi: 10.1016/j.csbj.2021.06.013 (PMC8258795; doi:10.1016/j.csbj.2021.06.013)
Supplement: Supplementary file 1 [file mmc1.pdf]

Appendix A. Supplementary Data

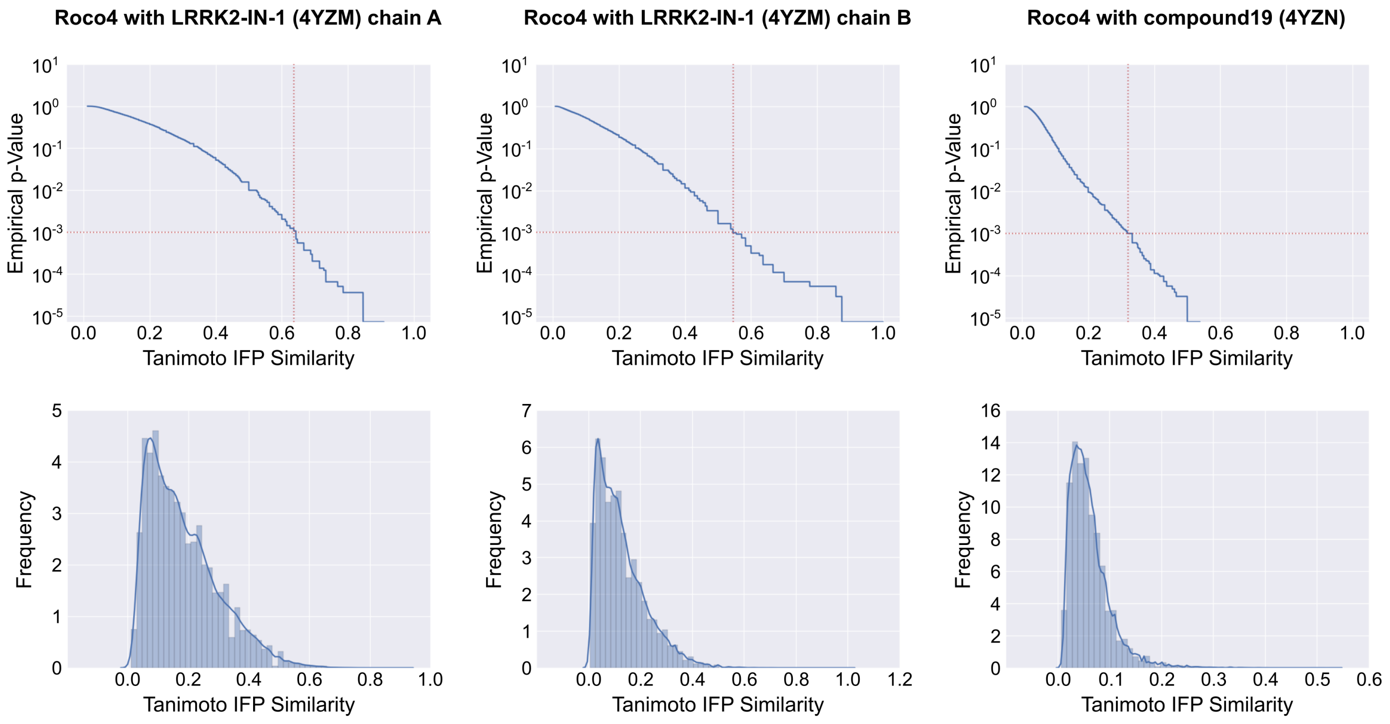

Figure A.1: Tanimoto interaction fingerprint (IFP) similarities and p-values of the virtual screening. Top, relation between Tanimoto IFP similarity and the empirical p-value for the screening results. Bottom, frequency of the Tanimoto IFP similarities.

Table A.1: The query and hit complexes of the structure-based virtual screening. The complexes are sorted by p-value. The annotation (Annot.) of the hits is FP for false positives, DC for hits that were discarded for another reason, and OK for hits that were pursued. UIDs denote the PDB ID, ligand ID, protein chain, and ligand position of the respective complex structure, in this order. UniProt of the hit protein, PubChem ID of the hit compound, and Tanimoto similarity of the query and hit interaction fingerprints are given. Notes give further information on the annotation.

Annot.	Query UID	Hit UID	Hit UniProt	Hit PubChem	Tanimoto	p-value	Notes
FP	4YZM:4K4:A:1301	4RRZ:LUR:C:705	Q05769	151166	0.846	7.20E-06	Lumiracoxib: Second hydrogen bond not visible. Patterns overall not well matching.
FP	4YZM:4K4:A:1301	4RRW:LUR:C:705	Q05769	151166	0.846	7.20E-06	Lumiracoxib (see above)
DC	4YZN:4K5:A:1301	3OID:TCL:C:604	P71079	5564	0.467	4.06E-05	Triclosan (preservative and antimicrobial). Not applicable for direct administration to humans. Used mainly in soaps and handwash.
OK	4YZM:4K4:B:1301	4L9I:8PR:B:601	P28327	43815	0.667	1.13E-04	Paroxetine: Has the two matching hydrogen bonds to different parts of the ligand.
OK	4YZN:4K5:A:1301	3VKX:T3:A:301	P12004	5920	0.364	3.00E-04	Liothyronine: Trinity of two halogen bonds and one hydrogen bond.
FP	4YZM:4K4:B:1301	4BZ7:B3N:B:701	A5H660	3994	0.625	3.23E-04	M344: Interaction patterns not matching well.
FP	4YZM:4K4:A:1301	5A6I:TCW:A:1124	P02766	4659569	0.667	5.47E-04	Tolcapone: Only one hydrogen bond. Otherwise just hydrophobic contacts.
OK	4YZM:4K4:A:1301	1XP0:VDN:A:201	O76074	110634	0.667	5.47E-04	Vardenafil: Patterns not entirely matching. But potential binding features are present in both compounds in the same fashion.
OK	4YZM:4K4:A:1301	4ANS:VGH:A:9000	Q9UM73	11626560	0.667	5.47E-04	Crizotinib: Very good interaction pattern agreement. Halogen groups are present at the right position, making a link to 4YZN.
FP	4YZM:4K4:A:1301	4D7B:TCW:A:1126	P02766	4659569	0.667	5.47E-04	Tolcapone (see above)
DC	4YZM:4K4:A:1301	3P50:PFL:D:320	Q7NDN8	4943	0.667	5.47E-04	Propofol: Used as sedative, side effects too severe for Parkinson's.
OK	4YZM:4K4:A:1301	1IKW:EFZ:A:2000	P03366	64139	0.643	6.77E-04	Efavirenz: Patterns not entirely matching. But matches well with the other query 4YZN (distances between functional groups).
OK	4YZN:4K5:A:1301	3MIY:B49:A:1	Q08881	5329102	0.333	9.98E-04	Sunitinib: Very good interaction pattern agreement.
FP	4YZM:4K4:A:1301	3B9M:SAL:A:1200	P02768	338	0.636	1.06E-03	Salicylic acid: Much too small. Not many matching features.
FP	4YZM:4K4:A:1301	4I89:1FL:A:201	P02766	3059	0.636	1.06E-03	Diflunisal: Too small. Not many matching features.
